# Five-canal maxillary first molar with calcified MB2 and low bifurcation of distal root canals: a case report

**DOI:** 10.3389/froh.2025.1511699

**Published:** 2025-03-28

**Authors:** Xuandong Lin, Hongyu Xie, Sijie Ruan, Xueling Ma, Jindong Long, Fangfang Xie

**Affiliations:** ^1^ College of Stomatology, Hospital of Stomatology, Guangxi Medical University, Nanning, Guangxi, China; ^2^ UWA Dental School, The University of Western Australia, Nedlands, WA, Australia; ^3^ Department of Anesthesiology, Central Hospital of Shaoyang, Shaoyang, Hunan, China

**Keywords:** rare root morphology, maxillary first molar, distobuccal 2, cone-beam computed tomography, case report

## Abstract

Root canal therapy is a highly regarded procedure, and failure to recognize the extremely variable anatomy of the maxillary first molars leads to unpredictable outcomes. This study reports the exceedingly rare case of a 27-year-old male with a maxillary first molar with one palatal and four buccal canals, and low bifurcation of the two distal root canals. The patient underwent nonsurgical endodontic treatment (root canal therapy), the buccal apical fistula and swelling disappeared completely, and imaging findings revealed a reduction of the apical shadow area. The patient did not experience a relapse during the 6-month follow-up period. This report suggests if thin root canal is not consistent with the root diameter, redundant root canals and low bifurcation should be considered. In maxillary first molars, it is important to find mesiobuccal 2 in the calcified root canal, still, using cone-beam computed tomography is essential for the exploration and evaluation of additional root canals, such as distobuccal 2, to prevent misdiagnosis.

## 1 Introduction

A distal root with two canals reflects a rare anatomy of the maxillary first molar. While maxillary first molars typically have three roots and three to four canals, the fourth canal is commonly found in the mesiobuccal (MB) root, rather than the distobuccal (DB) or palatal (P) root (1, 2). Root canal therapy (RCT) is a routine technique for treating pathological conditions of the maxillary first molars, such as infection or necrosis. Reasons for the failure of RCT and a retreatment rate as high as 19% are attributed to the inability to identify and negotiate missed canals (3). Therefore, root canal variations should be noticed during treatment, and related skills refined to appropriately address these natural variations (4). This paper describes the successful endodontic treatment of a maxillary first molar with five root canals, presenting with mesiobuccal 2 (MB2) and distobuccal 2 (DB2) roots with low bifurcation; this report describes a highly uncommon morphological variation associated with this tooth.

## 2 Case description

A 27-year-old male visited the Department of Prosthodontics at the Affiliated Stomatology Hospital of Guangxi Medical University due to porcelain chipping of the upper left posterior porcelain-fused-to-metal (PFM) crown and requested re-restoration. The patient reported that 7 years ago, he had undergone metal-ceramic crown restoration at a private dental clinic due to extensive dental tissue loss. Over the past 2 years, he occasionally experienced pain from thermal stimuli in the upper left posterior tooth, which had not been treated and gradually subsided on its own. In the past year, he had experienced gingival swelling with ulceration and pus discharge, but without spontaneous pain or discomfort on occlusion. One month ago, he noticed roughness when licking the upper left posterior tooth and experienced food impaction. Upon self-examination, he discovered the chipping of the crown. And there was no contributing medical history.

**Figure 1:**
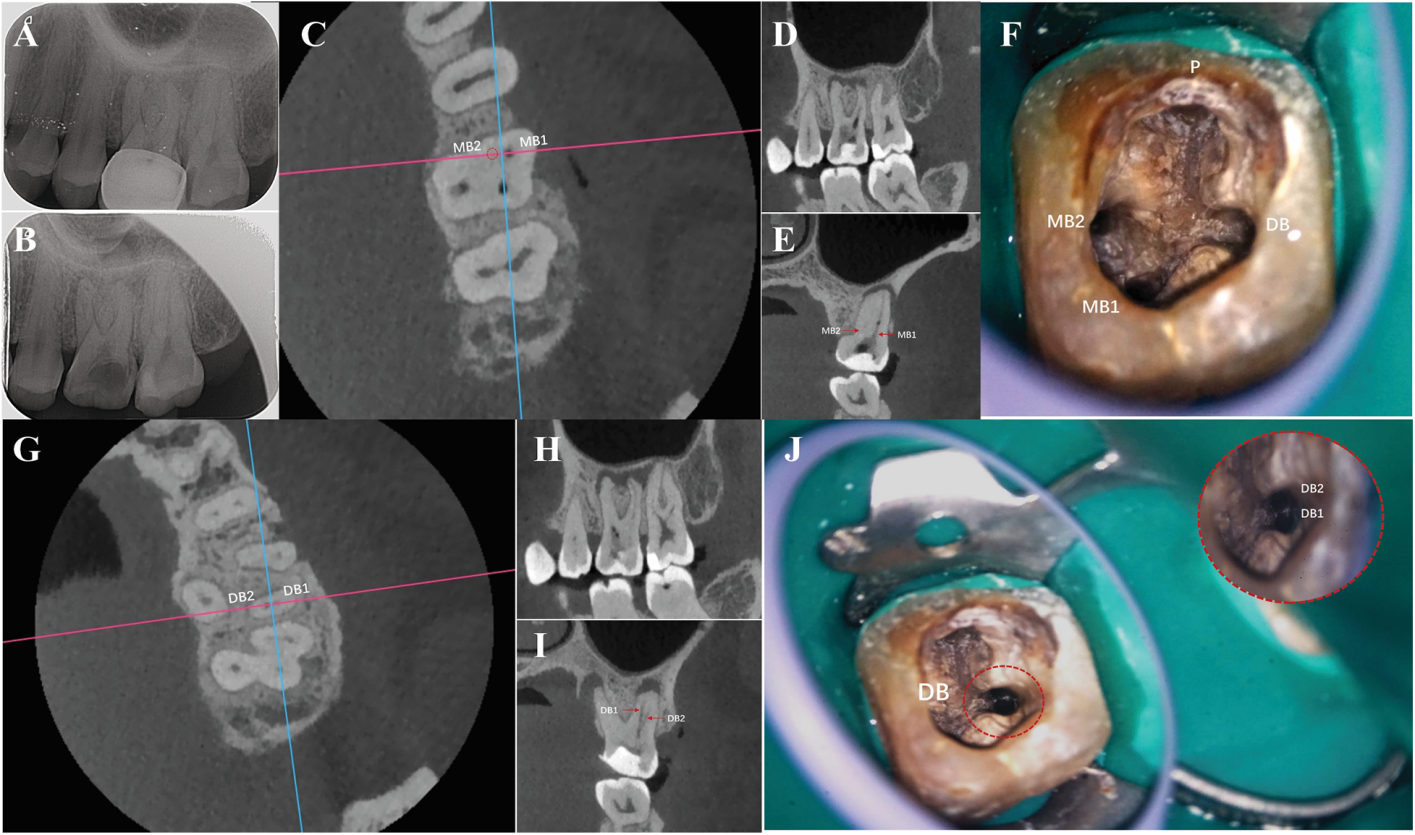
**(A,B)** Preoperative x-ray showing the maxillary first molar with irregular resorption of roots for periodic inflammation. **(C–E)** Cone-beam computed tomography (CBCT) image showing mesiobuccal 1 (MB1) suspectable mesiobuccal 2 (MB2), with calcifications sealing the coronal third of the root canal. **(F)** Isolated maxillary first molar with MB1, distobuccal (DB), palatal (P), and MB2 canals. **(G–I)** CBCT images showing that DB is only one root canal image with apical opening deviation; another DB canal may exist, and a low bifurcation in the apical third of the root; **(J)** low bifurcation after the enlarged upper end of distobuccal 2 (DB2) by the open file; inset is the DB canal, divided into DB1 and DB2.

Intraoral examination revealed exposure of chipped metal on the maxillofacial surface of a metal porcelain crown in tooth #26 with an improper margin fit. There was no response on percussion, but a gingival fistula and pulp exposure were present. Periapical radiography (Figures 1A,B) revealed a high-density blocked image of the crown restoration, no filling in the root canal, a periapical low-density area, and alveolar bone resorption. Therefore, pulp necrosis and chronic periapical periodontitis were diagnosed. The treatments proposed were: (1) to remove the porcelain-fused-to-metal crown restorations; (2) RCT by an endodontist; and (3) full-coverage zirconia crown restoration.

The patient was referred to our Endodontics department for the second stage of treatment. After further examination, a large soft-texture area of the buccal side of the occlusal surface on tooth #26 was found, with pulp exposure. No tenderness on probing or response to cold testing was observed. The number of electric pulp vitality tests was 80 (Parkell-Digitest®3, NY, USA). However, there was tenderness upon percussion in the apical region. Mobility was negative, and no deep periodontal pockets or cracks were detected. A fistula was visible on the buccal aspect, with pus discharge upon palpation.

After a thorough discussion about the treatment and its outcome, RCT was initiated for tooth #26 with patient's consent. Profound local anesthesia was achieved with one cartridge infiltration (Xylestesin-A 4%, Articaine 2 ml). Then rubber dam (Hygenic Elasti-Dam, OH, USA) isolation was performed, and previous fillings and caries were removed with the help of a round bur using a slow-speed handpiece (Dentsply Maillefer, Konstanz, Germany), after which the pulp chamber was opened, troughing was performed, and many calcifications were exposed. A dental operation microscope (DOM; Carl Zeiss Surgical, Oberkochen, Germany) was used to detect canals.

During the initial root canal exploration, a significant amount of calcification was found within the root canal system, and the MB2 canal was not detected. Considering the relatively high incidence of MB2 in the maxillary first molars (51.1%) among the Chinese population (5), we need more accurate imaging adjuncts to assess the root canal system in order to prevent missed canals. After obtaining informed consent, the patient was advised to undergo further examination with cone-beam computed tomography (CBCT; NewTom, Verona, Italy; Figures 1C–E). The settings (FOV and voxel resolution) were chosen for the patient based on the area to be examined and the diagnostic task in question. Considering the small FOV (6 × 6 cm, resolution 0.13-mm), the scan time was 23 s. Troughing and calcification removal were performed under an ultrasonic tip (Woodpex V, Guilin, China). Lateral perforation occurred during the search for MB2, and lateral wall repair was performed using iRoot BP plus (Innovative Bioceramix, Vancouver, Canada), and the MB, DB, P, and MB2 canals were negotiated (Figure 1F). The apical third of the DB root canal was thin, and the apical opening could not be directly detected. CBCT also revealed a deviation (Figure 1G), exposing the existence of another DB canal (Figure 1H). The low bifurcation was then detected (Figure 1I), therefore, the access opening was further modified in the coronal third of the root via endodontic access file (Yirui M3-Pro, Changzhou, China), and DB2 was successfully instrumented (Figure 1J).

A combination of electronic apex locators (Woodpex V, Guilin, China) and periapical radiographs was used to determine the working length (WL) of tooth #26: WL_MB_ = 20.0 mm, WL_MB2_ = 16.0 mm, WL_P_ = 18.0 mm, WL_DB_ = 19.0 mm, WL_DB2_ = 20.0 mm. Canals were then filled with nonsetting calcium hydroxide (Calcipulp, Saint-Maur, France), and the tooth was temporized (Cavit, Seefeld, Germany). After 1 week, the patient returned for the completion of nonsymptom-related treatment.

**Figure 2:**
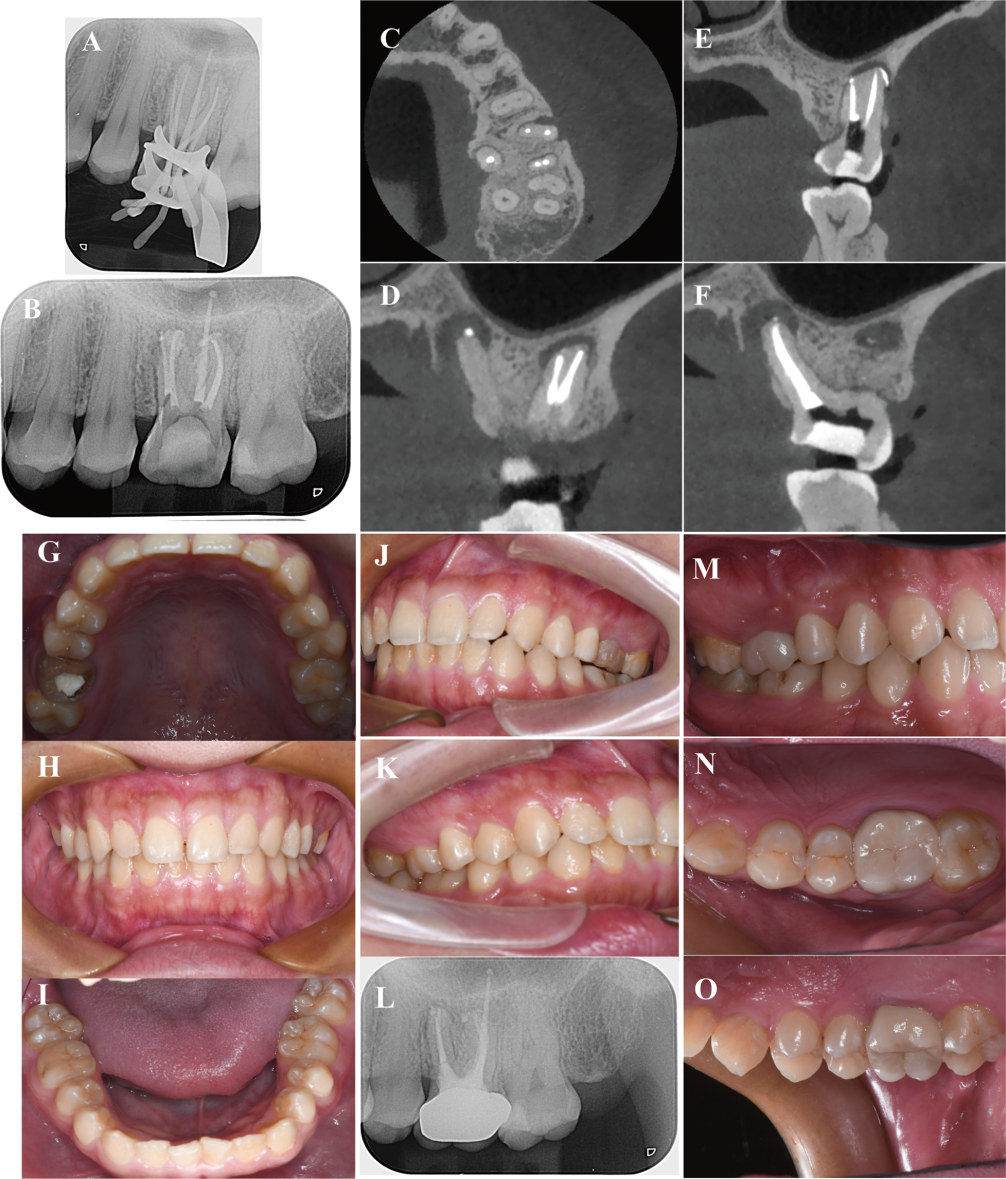
**(A)** Master cone radiograph showing the length in DB canals did not get to WL; **(B)** Postoperative X-ray shows root filling in place; **(C-F)** CBCT images of apical closure; DB and DB2 canals; MB and MB2 canals; P canal. **(G-K)** Digital images obtained 1-month postoperatively; the fistula and gingival erythema disappeared. **(L)** X-rays obtained 6 months postoperatively show that the area of the apical hypodense shadows reduced. **(M–O)** Digital images obtained at the 6-month follow-up.

All five canals of tooth #26 were then prepared and finished at an apical diameter of size 35# (MB1 0.04, MB2 0.04, DB1 0.04, DB2 0.04, P 0.06). The master apical file (MAF) for all root canals was 25#. Copious irrigation with 5% sodium hypochlorite was performed during the shaping and cleaning procedure. Trial radiography was performed, revealing that the length in the DB canals did not reach the WL; a jamming effect of the low bifurcation zone was therefore suspected (Figure 2A). Canal preparation was then modified to achieve the corrected length. The canals were dried with paper points, and single cone obturation was performed with iRoot SP (Innovative Bioceramix, Vancouver, Canada).

Subsequently, x-ray (Figure 2B) and CBCT examinations (Figures 2C–F) were performed to evaluate the root obturation of the five canals with various directions; each of the two DB (Figure 2D), two MB (Figure 2E), and P (Figure 2F) roots were correctly placed, and apical closure was achieved. To measure penetration, the cervical area of the proximal mesial tooth was presented as a high-density image of the cervical area of the tooth that was closed using iRoot BP Plus. One month postoperatively, the fistula and gingival erythema disappeared without significant percussion pain (Figures 2G–K). The outcome was successful, and the patient was advised to undergo a full-coverage zirconia crown restoration. At the 6-month follow-up, x-ray examination (Figure 2L) revealed that the extent of the apical hypodense shadows decreased, indicating that the inflammation was controlled, and bone regeneration occurred (Figures 2M–O).

## 3 Discussion and conclusions

Root canal variations refer to the abnormal changes in the number, shape, or structure of the root canal system. These variations can significantly affect the difficulty and outcome of root canal treatment. Research indicates that dental anatomy exhibits variations influenced by various factors, including ethnicity, age, and gender (6). The internal complexities of root canals are genetically determined and carry definitive importance in anthropology (7, 8). For instance, certain families or ethnic groups may be more prone to the presence of complex root canal morphology or additional root canals. A study on root canal variations in the Chinese population revealed that the incidence of additional canals in the distobuccal root (1.8%) and the palatal root (0.7%) (9) were partly similar to those observed in Burmese (10), Thai (11), and Korean populations (12), but lower than those in Indian populations (7). Additionally, studies have shown that age has a significant impact on the incidence of additional canals. As age increases, the additional canals decrease, primarily due to the natural narrowing of the pulp chamber and calcification of the root canal system (13). In this case, the age factor (27 years old) is an important reason for the complex variations in the root canal system.

In certain situations, such as dental trauma, auto-transplantation, or orthodontic treatment, the hard tissue deposition may be accelerated unexpectedly, leading to rapid narrowing or complete closure of the root canal space. This condition is referred to as calcific metamorphosis (CM), root canal calcification, or pulp canal obliteration (PCO). The incidence of complications during the treatment of calcified root canals is higher, including root canal deviation, ledges, instrument separation, and perforation (14, 15). Severely calcified teeth are susceptible to tooth perforation when entering the pulp chamber or initially locating the root canal opening, while adherent calcifications can impede the smooth passage of probes or other endodontic instruments through the pulp canal. Typically, the calcification process occurs in the apical direction from the crown; thus, root canal preparation tends to be easy once the root canal orifice calcification has been removed and the original root canal access has been found (16). However, in completely calcified root canal systems, locating the junction between the pulp chamber floor and the root development fusion line can be particularly difficult due to the dense calcifications that obscure anatomical landmarks. For maxillary molars, identifying the junction between the pulp chamber floor and the root canal wall, as well as tracing the dark developmental lines that outline the root canal perimeter, can help better detect the root canal orifice and thus avoid lateral perforation. Moistening the dentin can enhance the contrast between the grayish hue of the pulp chamber floor and the white tone of the secondary dentin, which aids in identifying the root canal orifice (17). Even with the assistance of a DOM, long-necked burs, and ultrasonic tips, highly experienced endodontists may still face challenges in creating an adequate access cavity and locating the root canal, which can lead to excessive loss of tooth structure and an increased risk of fracture and perforation (18). A new clinical method called “Guided Endodontics” has been developed to manage teeth with PCO. This method, which uses either static-guided (SG) or dynamic-guided (DG) techniques, offers an alternative way to prepare access cavities in the treatment of complex cases (19). In this case, the challenge in root canal exploration initially involved closure of the coronal third calcification. In comparison to perforation, the missing of root canals is considered to be even more unacceptable. Lateral perforation is a common complication encountered during the search for missed root canals and can be effectively managed using bio-ceramic materials such as iRoot BP or MTA. Empirical evidence indicates that the success rate of such repairs may reach as high as 72%–90% (20).

**Figure 3:**
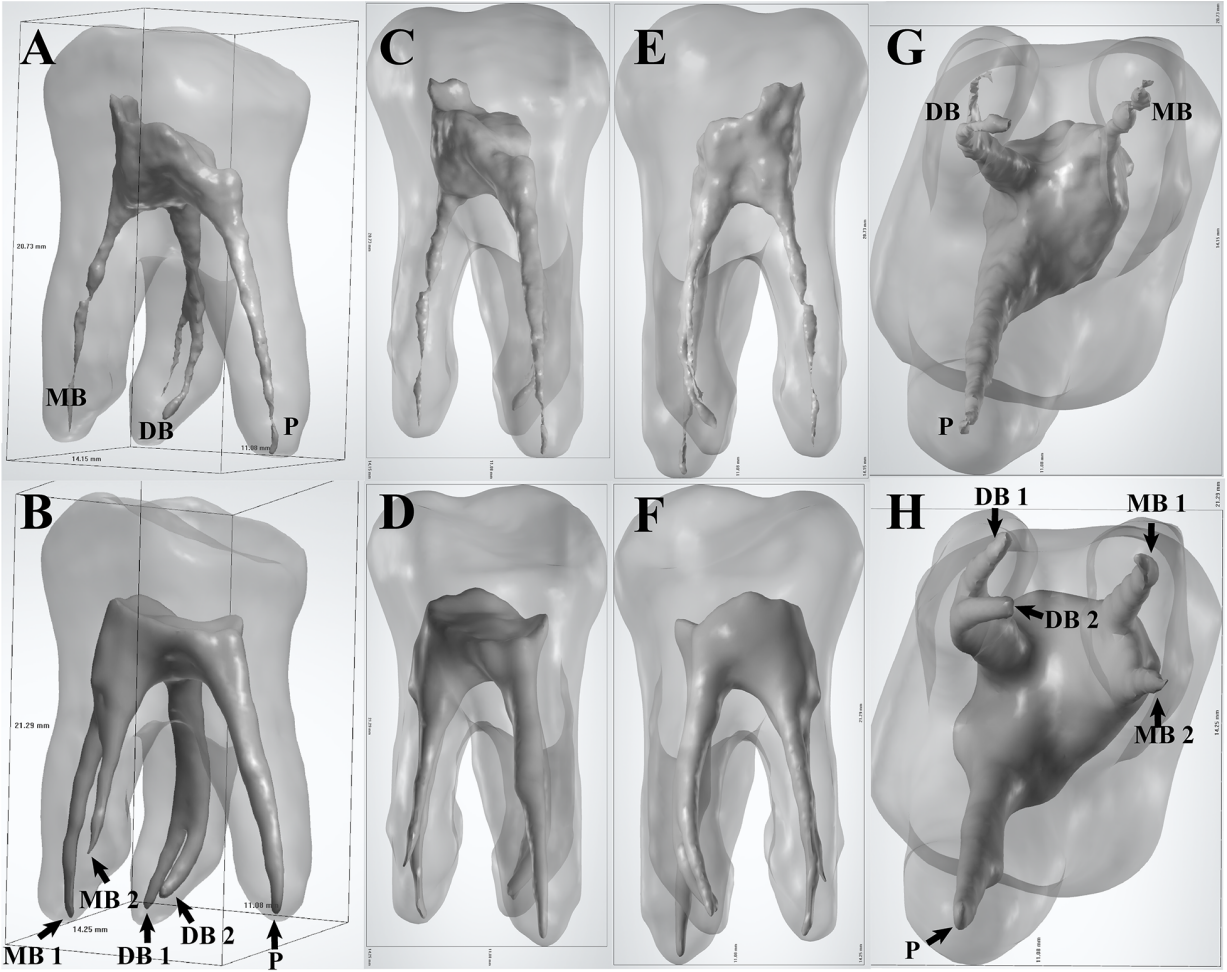
Three-dimensional modeling of the internal and external root canal morphology. **(A,C,E,G)** Before preparation; **(B,D,F,H)** after preparation.

Numerous studies and case reports in the literature demonstrate anatomical variations in human maxillary first molars. The prevalence of MB2 canals has a wide range owing to differences in the methodology of identification and race, with an average incidence of 73.8% (21). The relatively high incidence (81.27%–85.4%) of maxillary first molars among the Chinese population via CBCT makes searching for MB2 in the upper first molars a routine endeavor (22, 23). However, the inherent anatomical complexity and variability of root canal systems necessitate the occasional utilization of advanced diagnostic and therapeutic modalities, such as DOM, microendodontic instruments, and CBCT, to accurately discern and manage root canal variations. CBCT can clearly and comprehensively reflect the number, morphology, and curvature of root canals to preoperatively evaluate the difficulty of RCT, particularly in the maxillary molar region where periapical radiographs are sometimes difficult to interpret. A combination of three-dimensional images and microscopic ultrasound technology can therefore facilitate the precise implementation of RCT. However, in this case, due to the extensive diffuse modification (complete calcification) of the MB2 canal, the preoperative CBCT assessment was unable to reconstruct its three-dimensional image (Figure 3); thus, the experience of the practitioner is critical.

In addition to MB2, the detection rates of extra P and DB root canals of maxillary first molars are low, presenting with varying results depending on the population. For example, the incidences of DB2 were only 0.62%, 1.12%, and 1.25% among five root canals, while the incidences of P2 were only 0.31%, 1.76%, and 0.12% in the Brazilian, Chinese and Korean populations, respectively (12, 24, 25). The configuration of maxillary first molars with five root canals, with both MB2 and DB2, may reach an incidence of 1.2% (12). There are a few reports with complete case images depicting five canals with three roots; these are summarized in Table 1.

**Table 1:** Previous reports of five canals in maxillary first molars.

Root configuration	No. of canals	Root canal anatomy			Authors	Year
		MB	DB	P		
First maxillary molar						
3 roots	5	3	1	1	David B. Ferguson	(2005)
3 roots	5	3	1	1	Amauri Favieri et al.	(2006)
3 roots	5	1	1	3	Garg A. K. et al.	(2010)
3 roots	5	3	1	1	Mohammad Aminul Islam et al.	(2012)
3 roots	5	2	2	1	Jorge N.R. Martins et al.	(2013)
3 roots	5	2	2	1		
3 roots	5	2	2	1		
3 roots	5	2	1	2	Fahad Umer	(2014)
3 roots	5	2	2	1	Dakshita Joy Sinha et al.	(2016)
3 roots	5	2	2	1	Prem Anand	(2016)
3 roots	5	2	1	2	Kai Chen et al.	(2022)
3 roots	5	2	2	1	Chen Chen et al.	(2023)
3 roots	5	2	2	1	He Liu et al.	(2024)

DB2 canals are typically Type II (2-1) root canals, wherein two separate canals leave the pulp chamber and join just before the apex to form one canal, according to Vertucci's classification. However, this classification method can only describe the variations of a single root canal and cannot refine the number, position, and structure of the accessory canals. For the complex case described in this article, using the new classification method proposed by H. M. A. Ahmed (26, 27) may be more comprehensive, practical, and convenient. According to the new system of root canal morphology classification proposed by H. M. A. Ahmed, the root canal configuration of this case can be described as ^3^26 MB^2^DB^1−2^P^1^. The root canal type ^3^26 MB^2^DB^1−2^P^1^ is relatively rare in clinical practice. DB^1−2^ refers to a canal that divides into two separate and distinct canals near the apex, each with its own apical foramen; they are clinically known as low bifurcation root canals, making them more prone to omission and more difficult to manipulate. When addressing low-bifurcating canal anatomy, numerous challenges are encountered during the processes of canal preparation and obturation. Variability in Canal Configuration: Low-bifurcating canals often exhibit complex configurations, such as multiple canals that may merge or diverge at various levels. This anatomical variability complicates the accurate prediction and navigation of the canal anatomy. Visualization Challenges: Low-bifurcating canals are frequently located in areas with restricted visibility, making it difficult to identify and access all canal orifices. This limitation can result in incomplete cleaning and obturation of the canal system. Root Canal Orifice Access Design: In teeth with low-bifurcating root canals, the access to the root canals must be meticulously designed to ensure that instruments can fully access all root canals while preserving the structural integrity of the tooth as much as possible. In the present case, the DB canal demonstrated a low bifurcation. During the master cone fitting process, a “pseudo-binding” sensation was encountered due to the jamming effect. This phenomenon could potentially lead to the master cone not reaching the WL. To circumvent this issue, sequential obturation of each canal was performed. This method not only achieved tight and complete obturation but also minimized the removal of tooth structure at the canal orifice.

This narrative provides the necessary strategy for the management of calcified and variant canals. This consistent strategy is based on the following principles: diagnosis of endodontic and periapical disease, preoperative imaging assessment of the anatomical structures of the pulpal system, personalization of the pulpal access, exploration of the pulpal cavity under the DOM, use of ultrasound to locate and remove calculi, and safe enlargement of the calcified and variant canals with highly flexible and fatigue-resistant instruments (28, 29).

## Data Availability

The original contributions presented in the study are included in the article/Supplementary Material, further inquiries can be directed to the corresponding author.
